# A. V. M. A.

**Published:** 1899-06

**Authors:** 


### A. V. M. A.

It is hoped that all members who anticipate joining in the discussion of this topic will review their papers and give them that full and free criticism which their importance merits.

Another intersting topic. A paper is announced from the pen of Dr. Leonard Pearson on the subject "The Suppression of Tuberculosis in Pennsylvania." While much has been written relative to tuberculosis, the subject was never more alive than at the present time nor the outlook more favorable for securing public support for the suppression of this disease. As no State has made more progress and dealt more conservatively with this subject, it will be one of special importance as to future work in this direction.

Dr. M. H. Reynolds will offer some "Notes on the Healing Process in Ovariotomy." The doctor has given this subject considerable study and made many fine photographs of dissections illustrating the points made in his paper.

Dr. W. H. Dalrymple will present a very important paper at the meeting this year on the subject of "Dietetics," a subject to which the average practitioner gives very little attention.

"The Treatment of Azoturia" is the title of a paper to be presented by Dr. Richard P. Lyman, of Hartford, Conn. Every practitioner must take a lively interest in this subject because of the fatality of this disease, especially during certain seasons and in some localities.

Perhaps no topic will prove more interesting than one to be presented by Dr. E. A. A. Grange, Dean of the Veterinary Department of the Detroit College of Medicine and late State Veterinarian for Michigan, "Disinfection." The doctor will demonstrate some agents and methods. The difficulties which confront the veterinary surgeon and veterinary sanitarian in the practical application of disinfectants have led many to assert that the use of such agents must be very limited. The paper and its discussion will doubtless help to make the problem less difficult. Dr. Grange offers to furnish to any member one of the copies of his paper, in order that those who would like to specially discuss and be more enlightened on the same in advance by giving the points established in the paper.

The railroads entering New York City have granted a reduction in rates for our meeting, giving us a one and one-third fare for the round trip, on the certificate plan, and the time-limit of the meeting is from September 4th (Monday) to September 9th (Saturday), inclusive, with three days before and after for the journey to and from New York City. It is probable that a similar reduction will be granted by all connecting railway associations.

Dr. S. B. Nelson, of Pullman, Washington, State Veterinarian, writes that he expects to attend the meeting in New York, and hopes to secure the attendance of other veterinarians from the Pacific slope.

It is probable that a Massachusetts member will contribute a paper for the coming meeting on the subject of "Rabies." The paper will include a series of observations and special studies.

Dr. E. B. Ackerman, of Brooklyn, N. Y., who had a large experience in municipal inspection, will open the discussion on "Municipal Meat-inspection," basing his remarks on the papers presented at the Omaha meeting.

